# Point-of-Care Method T2Bacteria^®^Panel Enables a More Sensitive and Rapid Diagnosis of Bacterial Blood Stream Infections and a Shorter Time until Targeted Therapy than Blood Culture

**DOI:** 10.3390/microorganisms12050967

**Published:** 2024-05-11

**Authors:** Tamara Clodi-Seitz, Sebastian Baumgartner, Michael Turner, Theresa Mader, Julian Hind, Christoph Wenisch, Alexander Zoufaly, Elisabeth Presterl

**Affiliations:** 1 Department of Infectious Diseases and Tropical Medicine, Klinik Favoriten, 1100 Vienna, Austria; 2 Department of Rheumatology and Osteology, Klinik Favoriten, 1100 Vienna, Austria; 3 Faculty of Medicine, Sigmund Freud University Vienna, 1020 Vienna, Austria; 4 Department of Hospital Epidemiology and Infection Control, Medical University of Vienna, 1090 Vienna, Austria

**Keywords:** T2Bacteria^®^Panel, T2 magnetic resonance, bloodstream infection, culture-independent diagnostics

## Abstract

Background: Rapid diagnosis and identification of pathogens are pivotal for appropriate therapy of blood stream infections. The T2Bacteria^®^Panel, a culture-independent assay for the detection of *Escherichia coli*, *Enterococcus faecium*, *Staphylococcus aureus*, *Klebsiella pneumoniae*, *Acinetobacter baumannii*, and *Pseudomonas aeruginosa* in blood, was evaluated under real-world conditions as a point-of-care method including patients admitted to the internal medicine ward due to suspected blood stream infection. Methods: Patients were assigned to two groups (standard of care—SOC vs. T2). In the SOC group 2 × 2 blood culture samples were collected, in the T2 group the T2Bacteria^®^Panel was performed additionally for pathogen identification. Results: A total of 94 patients were included. Pathogens were detected in 19 of 50 patients (38%) in the T2 group compared to 16 of 44 patients (36.4%) in the SOC group. The median time until pathogen detection was significantly shorter in the T2 group (4.5 h vs. 60 h, *p* < 0.001), as well as the time until targeted therapy (antibiotic with the narrowest spectrum and maximal effectiveness) (6.4 h vs. 42.2 h, *p* = 0.043). Conclusions: The implementation of the T2Bacteria^®^Panel for patients with sepsis leads to an earlier targeted antimicrobial therapy resulting in earlier sufficient treatment and decreased excessive usage of broad-spectrum antimicrobials.

## 1. Introduction

Bloodstream infections (BSIs) cause significant morbidity and mortality in patients all over the world [1,2]. A delay in diagnosis and appropriate therapy increases mortality significantly [2,3]. *Escherichia coli*, *Staphylococcus aureus*, *Klebsiella pneumoniae*, and Streptococcus pneumoniae are found to be the most common causing pathogen of community-acquired BSIs [4,5]. In hospital-acquired or hospital-associated infections apart from *Escherichia coli*, *Staphylococcus aureus*, *Klebsiella pneumoniae*, *Enterococcus* spp., *Pseudomonas aeruginosa*, and *Acinetobacter baumanii* are the most common causative pathogens [5].

The present gold standard to detect BSI is collecting blood cultures (BCs). However, growth and identification of the pathogen using BCs can take up to 24 h or even longer [1].

There have been many promising attempts to increase the speed of pathogen detection in positive BCs using culture-independent methods. Several PCR-based tests (e.g., BioFire^®^ FilmArray Blood Culture Identification from Biomerieux in Salt Lake City, UT, USA and Luminex^®^ VERIGENE^®^ from DIASORIN in Saluggia, Italy), matrix-assisted laser desorption ionization–time of flight mass spectrometry (MALDI-TOF), or Loop-Mediated Isothermal Amplification (LAMP) based tests performed on a positive blood culture have shown to reduce pathogen detection time and optimize the antibiotic regimen [6,7,8,9]. The disadvantages of these methods are the requirement of a positive blood culture as well as the know-how and availability of the microbiology lab [5]. Next-generation sequencing (NGS) presents a promising method for diagnosing BSI, enabling shorter duration and higher sensitivity in pathogen detection compared to culture-based methods [10,11]. However, as of yet it is not suitable for general use due to its high cost and the specific knowledge and training required.

Recently another culture-independent assay for the detection of bacteremia and candidemia was launched. These innovative tests use the so-called T2 magnetic resonance (T2MR) technique, which can detect minimal amounts of pathogens in whole blood samples in 3–5 h [4]. T2MR lyses red blood cells, concentrates microbial cells and cellular debris, lyses cells by mechanical bead-beating, amplifies DNA using a thermostable polymerase and target-specific primers, and detects amplified products by amplicon-induced agglomeration of supermagnetic partiles and T2MR measurement. Two panels using this method are currently available, one for the detection of five different Candida species (T2Candida^®^Panel) and the other for the detection of *Escherichia coli*, *Enterococcus faecium*, *Staphylococcus aureus*, *Klebsiella pneumoniae*, *Acinetobacter baumannii*, and *Pseudomonas aeruginosa* (T2Bacteria^®^Panel) in blood. These pathogens are summarized with the abbreviation ESKAPE.

Several studies demonstrated a high sensitivity and specificity of T2Candida^®^Panel [12,13,14,15,16] and decreasing health-care costs in patient populations with an increased risk for candidemia [17], as well as a high sensitivity and specificity of T2Bacteria^®^Panel [18].

However, there are no data so far about the clinical impact of the use of T2MR in patient’s outcomes.

To address this knowledge gap, we performed a prospective study to evaluate the usability and effect of T2Bacteria^®^Panel in patients with suspected bacteremia by using either both T2Bacteria^®^Panel and BC or BC alone as the diagnostic standard of care. The main objective was to evaluate whether the implementation of the T2Bacteria^®^Panel compared to blood culture for the detection of BSI enables an earlier targeted therapy.

The secondary aim was to compare sensitivity of T2Bacteria^®^Panel and BC and the time until positive or negative result. Lastly, we wanted to evaluate the potential clinical benefit of using T2Bacteria^®^Panel for patient outcomes such as mortality and length of hospital stay.

## 2. Materials and Methods

### 2.1. Patients

The study was performed from 17 November 2018 to 14 February 2020 at the Department of Infectious Diseases of the Klinik Favoriten hospital in Vienna. The hospital consists of 17 different departments with a total of 781 beds. The emergency unit is open 24 h a day, seven days a week. Patients with signs and symptoms of infection with need of hospitalization are transferred to the Department of Infectious Diseases any time of day. This department provides 72 normal ward patient beds and ten intensive care unit beds.

The study included patients (aged > 18 years) admitted to the department with high suspicion of BSI caused by an ESKAPE pathogen. To include the patient, signs of systemic infection and bacteria specific clinical signs/symptoms of infection with an ESKAPE pathogen must be present. The exact inclusion and exclusion criteria are seen in the Supplementary Material (Supplement S1).

### 2.2. Study Design and Methods

Every potential study participant was informed about the procedures and objectives of the study. Upon agreement, a consent form was signed by the patient. Participants characteristics (age, sex, Charlson Comorbidity Index) and clinical parameters ((temperature measured in ear, duration of fever, Hospital Recovery Score (HRS) at admission)) were collected.

Study participants were split into two groups depending on the day of admission (even vs. uneven day). Patients admitted on the even days of the month were allocated in group “T2 Panel (T2)”, patients admitted on uneven days in group “Standard of Care (SOC)”.

Blood samples for the diagnostic standard procedure (2 aerobic and 2 anaerobic BC bottles, blood count and inflammation parameter CRP) were taken for each patient in both groups and were collected prior to initiation of antimicrobial therapy. BC collection and the identification of pathogens isolated from positive blood culture bottles was performed according to routine procedures. Negative results were defined as no growth of bacteria in seven days.

In the group “T2” EDTA blood was subjected to analysis on the T2MR (located at the department) using T2Bacteria^®^Panel according to manufacturer’s instructions immediately following blood sampling by the physician in charge of the patient.

Antibiotic regimens were selected empirically based on suspected pathogens and were switched once a pathogen was detected in BC or T2Bacteria^®^Panel following the guidelines for targeted therapy of bacterial infections in adults of the Paul-Ehrlich-Gesellschaft für Infektionstherapie [19].

The participants were followed up at time of discharge or death. The following parameters were evaluated:

- In-hospital mortality
- Need of mechanical ventilation, vasopressors or Continuous Renal Replacement Therapy
- Length of hospital stay
- Change of HRS at admission and day 7 of hospital stay
- Length of clinical recovery
- Time until positive or negative result using BC and T2MR
- Frequency and time until targeted therapy

Following the guidelines of Paul-Ehrlich-Gesellschaft für Infektionstherapie [20], targeted therapy was defined depending on the detected pathogen (without knowledge of resistances) as seen in Supplement S2.

If antimicrobial resistance was present, targeted therapy was defined as the use of an antibiotic with the narrowest spectrum and maximal effectiveness based on the antibiogram.

In the case of pathogen detection in only T2Bacteria^®^Panel or BC, we applied “true-infection criteria”, similarly to prior studies [20,21]. If the detected pathogen was isolated in a sample type from the suspected focus, such as urine or tracheal secretion, the result was regarded as true positive. If sampling of a different sample type was not possible at least two different infectious diseases specialists evaluated the results and if they agreed that the detected pathogen reflected the focus of and fit the nature of the underlying infection, the result was also regarded as true positive.

### 2.3. Statistical Analysis

Categorical variables are described using absolute numbers and percentages. For continuous variables mean and standard deviation were calculated. In case of highly skewed variables quartiles are reported instead of mean and standard deviation.

Comparisons between BC and T2 regarding continuous variables were performed with t-tests for independence sample and Welch correction in case of heterogeneous variances. In cases with highly skewed distribution of outcome values, Mann–Whitney-U-tests were performed. Regarding categorical outcome variables, chi-square tests were used and Fisher’s exact test in cases with small expected counts. *t*-test for dependent variables was used for testing differences within patients. A type I error rate of 5% was used for inference. SPSS (v29.0.2) and STATA (v18) were used for all statistical analyses.

#### Sample Size

Initially we planned to enroll at least 200 patients. Since it was a pilot study, an initial sample size calculation was not possible; an interim analysis was planned to determinate the exact sample size. However, the study was terminated prematurely due to the COVID-19 pandemic beginning in Austria in early 2020. Given that only patients suffering with COVID-19 were admitted to the hospital, the inclusion criteria could not be met. We acknowledge that a higher number of included patients would have increased the statistical power of the study. Regardless, not knowing how long the pandemic would last, we decided to terminate the study and present the collected data.

### 2.4. Compliance with Ethical Standards

A written agreement was obtained from all participants. The study was performed in accordance with the Declaration of Helsinki, the project plan was presented to and approved by the ethics committee of City of Vienna (18-190-0918).

The T2 instrument as well as the necessary test kits were provided by T2Biosystems through Biomedica (Vienna, Austria). There was no financial support, there were no conflicts of interests.

## 3. Results

**Figure 1:**
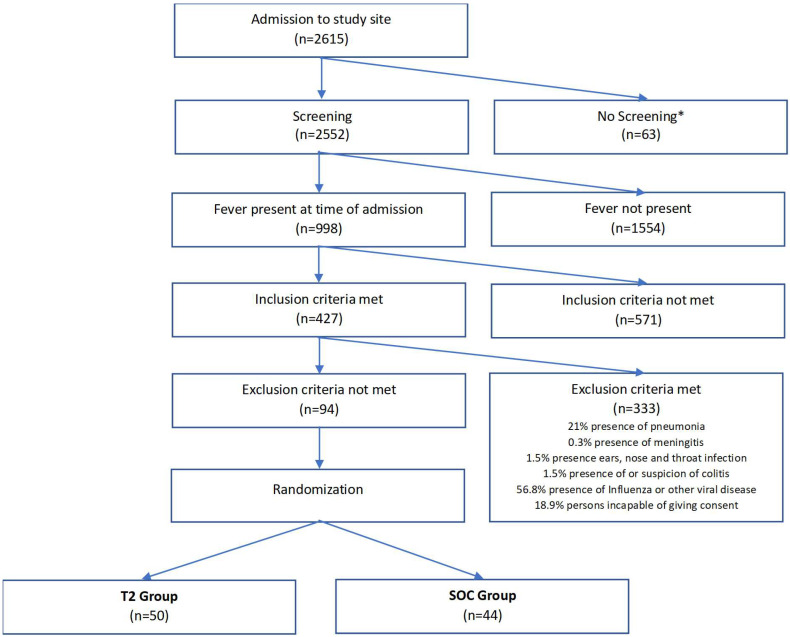
Study flow. * no screening due to lack of personal resources.

Overall, 94 patients (46 male, 48 female) were enrolled. As seen in Figure 1, during this time period 2615 patients were admitted to the study site (2388 to normal wards and 227 to Intensive Care Unit).

After applying inclusion and exclusion criteria, 94 patients were included in the study—50 patients in total were included in the T2 group and 44 patients in the SOC group.

The basic parameters of the two groups are stated in Table 1. There are no statistically significant differences in the groups.

The clinical parameters at time of study inclusion are seen in Table 2. Again, there was no significant difference in the T2 and SOC group.

### 3.1. Comparison T2 and BC Group

As seen in Table 3, an ESKAPE pathogen was detected in 17 of 50 (34%) patients in the T2 group, two extra-panel pathogens were isolated by BC, increasing the pathogen detection rate to 38%. In 44 of 50 patients, at least one alternative culture (surrogate) of a different sample type was performed. In 9 of the 17 patients (52.9%) the pathogen detected in T2Bacteria^®^Panel could be cultured in a different sample as well (7 times detected in urine, 1x in bronchoalveolar lavage, 1x wound swab). In the remaining cases, all positive results of the T2Bacteria^®^Panel were considered true positive as well. There were no cases of negative T2Bacteria^®^Panel results and simultaneous detection of ESKAPE pathogen in BC or culture of a different sample type. No multi-drug resistant pathogen was detected.

In SOC group an ESKAPE pathogen was detected in 20.5% of the patients (6x *E. coli*, 2x *S. aureus*, 1x MRSA), seven extra-panel pathogens were isolated by BC (3x *S. pneumoniae*, 2x *P. mirabilis*, 1x *E. faecalis*, 1x *P. multocida*), increasing the pathogen detection rate to 36.4%.

### 3.2. Antimicrobial Therapy

**Figure 2:**
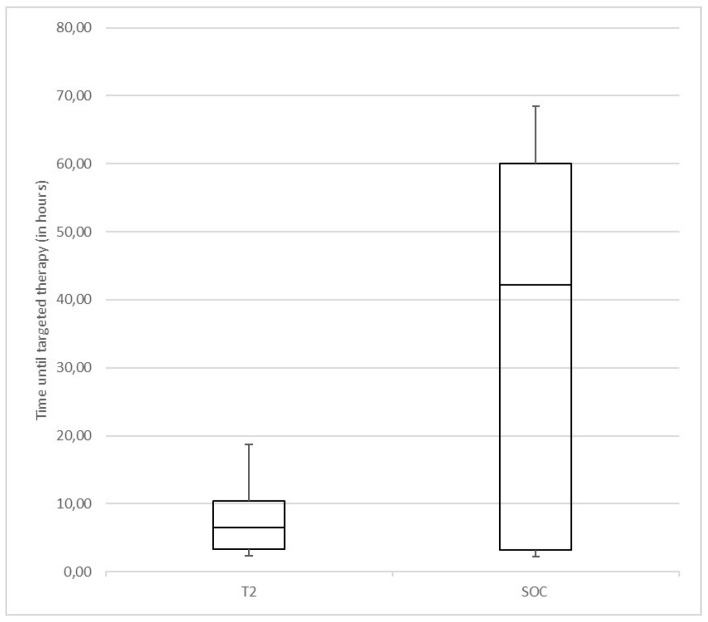
Time until targeted therapy in T2 group (using T2Bacteria^®^Panel and BC) and SOC group (using BC only).

As seen in Figure 2, the time until targeted therapy since hospital admission was significantly shorter in the T2 group (median 6.4 h, range: 0.6–18.7 h) than in the BC group (median 42.7 h, range: 3.5–68.5 h) (*p* = 0.043).

### 3.3. Clinical Outcome

There was no significant difference regarding in-hospital mortality ((T2 2% (1/50) vs. BC 2.3% (1/44), *p* = 1)), need of mechanical ventilation (0/50 vs. 0/44), vasopressors ((8% (4/50) vs. 2.3% (1/44), *p* = 0.357)) or Continuous Renal Replacement Therapy ((2% (1/50) vs. 0% (0/44)), *p* = 1.000, length of hospital stay ((T2 mean 10.8 days (SD 6.6) vs. BC 13.4 days (SD 10.1), *p* = 0.169)), change of HRS at admission and day 7 of hospital stay (T2 44% equal, 56% improvement, 0% deterioration vs. BC 50% equal, 50% improvement, 0% deterioration; *p* = 0.561) or length of clinical recovery ((T2 mean 3.2 days (SD 2.2) vs. BC 3.8 days (SD 3.4), *p* = 0.322)).

### 3.4. Time to Result Comparison between T2Bacteria^®^Panel and BC

Evaluation of the 50 patients in the T2 group only enables a direct comparison of T2Bacteria^®^Panel and BC. As seen in Table 3, T2Bacteria^®^Panel detected more pathogens than BC (17 of 50 ESKAPE pathogens vs. 7 of 50). As mentioned above, all positive results of T2Bacteria^®^Panel were considered true positive.

**Figure 3:**
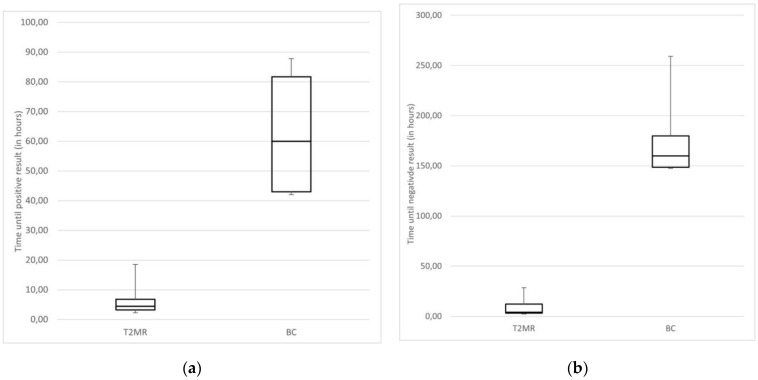
(**a**) Time until positive result (in hours) using T2Bacteria^®^Panel and BC; (**b**) time until negative result (in hours) using T2Bacteria^®^Panel and BC.

The time until detection of pathogen since blood drawn and positive (median 4.5 h vs. 60 h, *p* < 0.001) and negative result (median 4.2 h vs. 160.2 h, *p* < 0.01) is significantly shorter with the T2Bacteria^®^Panel compared to BC, as seen in Figure 3a,b.

## 4. Discussion

This study shows that additional use of T2Bacteria^®^Panel with T2MR and BC during admission of patients with high risk of BSI led to a more frequent and earlier detection of the causative pathogen and therefore targeted therapy compared to the use of BC alone.

Using T2Bacteria^®^Panel and BC resulted in a significantly shorter time until sufficient therapy in the T2 group. In 7.4% of the included patients in our cohort, the empiric antimicrobial therapy did not cover the causative pathogen. Given that increasing time until appropriate antimicrobial therapy is accompanied by longer hospital stay and lower survival rates [2,3,22], these findings are promising. In 10.6% of the included patients, a de-escalation was possible, again significantly earlier in the T2 group than in the SOC group. Given that prolonged use of broad-spectrum antimicrobials is not only accompanied with a higher risk of side effects for the individual patient, but also a known risk factor for development and spread of antimicrobial-resistant microorganisms, this aspect demonstrates a further advantage of the use of the T2Bacteria^®^Panel.

Several studies have shown the clinical benefits of rapid molecular diagnostic tests for bloodstream infections (BSIs). For instance, Tafelski et al. and Gies et al. found that PCR-based tests like LightCycler^®^ SeptiFast detect pathogens more sensitively and identify them faster than blood culture [23,24]. Similarly, Box et al. evaluated Luminex^®^ VERIGENE^®^ Gram-Positive Blood Culture, which detects various pathogens and resistance genes within two hours of positive blood culture. They found a significant reduction in time until targeted therapy after implementation [25].

Felsenstein et al. also observed a decrease in time until targeted therapy in patients with Gram-positive BSI [26]. Eby et al. combined Luminex^®^ VERIGENE^®^ with infectious diseases consultation, resulting in even shorter time to therapy initiation, especially for methicillin-sensitive *S. aureus* [27].

Weng et al. assessed MALDI-TOF implementation, showing reduced mortality, faster pathogen detection, and shorter hospital stays post-implementation [28]. Similarly, Cavalieri et al. found shorter time until pathogen identification, decreased hospital stay, and reduced antimicrobial therapy duration after MALDI-TOF implementation [29].

Lastly, Wang et al. investigated NGS, which led to more frequent pathogen identification post-implementation, reducing the need for debridement and antimicrobial therapy, lowering antibiotic-related complications, and decreasing the cost of antimicrobial therapy [30].

Even if these results seem promising, the disadvantages of these methods are the necessity of a positive blood culture as well as the expertise and availability of the microbiology lab [5].

Further, the limited comparability due to the design of these studies is a huge limitation.

In our study we used the T2Bacteria^®^Panel as a point-of-care method 24 h a day to overcome the disadvantage of being highly dependent on a microbiology lab. The study design was prospective and pseudo-randomized.

The fact that we used the T2Bacteria^®^Panel as a point-of-care method enables the remarkable short median time of 6.4 h until targeted therapy after patient enrolment into our study. Similar to our study is the one from Voigt et al. [31] evaluating T2Bacteria^®^Panel as a point-of-care method at the emergency department. There, T2Bacteria^®^Panel was performed in 137 adults presenting at the emergency department. The results were comparable to ours, showing that time until targeted therapy could be reduced, yet only in eight patients. An explanation for the small number is that the rate of positive results within the study was much lower than ours, due to lack of predefined inclusion criteria. Every time a BC was ordered from the physician in charge, T2Bacteria^®^Panel was performed as well. We propose implementation of specific criteria on when to use T2Bacteria^®^Panel to avoid excessive numbers of negative results, which ultimately increase costs and workload. Only patients highly susceptible to BSI caused by ESKAPE pathogens were included in our studies and the positivity rate of T2Bacteria^®^Panel was 34%. In comparison, had BC been used alone, a pathogen would have been found in 18% of the cases.

It is important to underline that T2Bacteria^®^Panel should only be used in conjunction with BC or samples from other sites (e.g., respiratory secretion) since T2MR firstly can only detect a limited number of pathogens and secondly can only detect them in blood. Further, BC offers the possibility of antibiotic resistance testing. To overcome this disadvantage, T2MR offers a T2Resistance^®^Panel [32], which can detect antibiotic resistance genes. However, this panel was not evaluated in this study. Another disadvantage of T2Bacteria^®^Panel are the higher costs compared to BC. However, by using T2Bacteria^®^Panel as a point-of-care method, and therefore not requiring professional laboratory and additional personnel, costs could be reduced. The decreased usage time of broad-spectrum antibiotics might reduce costs as well. This aspect should be addressed in future studies.

### Limitations

The largest limitation is the small sample size, making the study underpowered regarding the impact of outcome parameters. Nevertheless, a prior comprehensive literature research showed that the utilization of rapid molecular techniques such as PCR-based methods, MALDI-TOF, or NGS not only reduce time until pathogen detection and targeted therapy, but also significantly reduce length of hospital stay and mortality compared to BC utilization alone [1,25,26,27,28,29,30,31]. A similar effect may be presumed with T2Bacteria^®^Panel. Therefore, further studies with larger study samples are necessary to evaluate this aspect. Another limitation is that we only performed a pseudo-randomization (admission at even vs. uneven days). It is important to add that even if we compared the T2MR results with cultures of other samples and evaluated whether the detected pathogen fits the clinical presentation and risk factors of the individual patient prior to decide whether the result is true positive, false positive results of T2MR cannot be fully excluded. In previous studies the rate of false positive results with T2MR was reported between 0–28.5% [14,18,33] depending on the criteria for “true infection” used. In our opinion, in some of the studies the false positive rate is overrated. However, until we have more robust clinical data, it is advisable to critically evaluate each result of T2MR and verify whether the detected pathogen corresponds to the patient’s clinical presentation and suspected focus.

## 5. Conclusions

The implementation of T2Bacteria^®^Panel in patients with suspected BSI leads to an earlier targeted antimicrobial therapy resulting in earlier sufficient treatment and decreased excessive usage of broad-spectrum antimicrobials. A reduction in side effects and risk of resistance development can be presumed.

## Figures and Tables

**Table 1:** Basic parameters in T2 and SOC group.

	T2 (n = 50)	SOC (n = 44)	*p*-Value
**Sex**			*p* = 0.295
*female (%)*	*23 (46)*	*25 (56.8)*	
*male (%)*	*27 (54)*	*19 (43.2)*	
**Median age in years (min–max)**	*69.00 (19–95)*	*71.50 (30–94)*	*p = 0.178*
**Median** **Charlson Comorbidity Index (** **min–max** **)**	*4 (0–10)*	*5 (0–11)*	*p = 0.404*
**Suspected infection at admission**			
*Urine tract infection (%)*	62 (31)	59.1 (26)	
*Skin infection (%)*	20 (10)	27.3 (12)	
*Signs of abscesses (%)*	0 (0)	0 (0)	
*Clinical suspected spondylodiscitis (%)*	2 (1)	6.8 (3)	
*Recent abuse of IV-Drugs (%)*	4 (2)	2.3 (1)	
*Endocarditis (%)*	2 (1)	2.3 (1)	
*Presence of intravascular devices (%)*	2 (1)	0 (0)	
*Presence of peritoneal dialysis (%)*	0 (0)	0 (0)	
*Colonization with ESKAPE spp. (%)*	4 (2)	4.5 (2)	
*Intraabdominal infection (%)*	8 (4)	9.1 (4)	

SD = Standard deviation, qSOFA = Quick Sequential Organ Failure Assessment, CRP = C-reactive protein, WBC = white blood cells, IV = Intravenous.

**Table 2:** Clinical parameters at time of hospital admission in the group T2 and SOC.

	T2 (n = 50)	SOC (n = 44)	*p*-Value
**Temperature measured in ear (in °C)**			
*Mean (+/−SD)*	38.7 (0.55)	38.6 (0.57)	*p* = 0.315
**Duration of fever (in days)**			
*Mean (+/−SD)*	2.3 (1.56)	2.4 (3.01)	*p* = 0.851
**HRS (in %)**			
*2 points*	72 (36)	70.5 (31)	
*3 points*	16 (8)	25 (11)	*p* = 0.340
*4 points*	12 (6)	4.5 (2)	
**qSOFA (in %)**			
*0 points*	56 (28)	45.5 (20)	
*1 points*	30 (15)	38.6 (17)	*p* = 0.583
*2 points*	14 (7)	15.9 (7)	
**CRP (mg/l)**			
*Mean (+/−SD)*	165.1 (116.7)	139.4 (101.1)	*p* = 0.260
**WBC (G/L)**			
*Mean (+/−SD)*	13.6 (5.7)	13.8 (5.9)	*p* = 0.814

HRS = Hospital Recovery Score (1 point = Not hospitalized, 2 points = Non-ICU hospitalization, not requiring supplemental oxygen, 3 points = Non-ICU hospitalization, requiring supplemental oxygen, 4 points = Admitted to the ICU, not requiring invasive mechanical ventilation, 5 points = Requiring invasive mechanical ventilation, 6 points = Death); qSOFA = Quick Sequential Organ Failure Assessment (breathing rate ≥ 22/min, Glasgow Coma Score < 15, systolic blood pressure ≤ 100 mmHg); CRP = C-reactive protein; WBC = white blood count.

**Table 3:** T2 group: Results of T2Bacteria^®^Panel and BC in compared to culture of sample of suspected focus (surrogate).

Species	T2Bacteria^®^Panel (n)	BC +	BC −	Surrogate +	Surrogate −
*E. coli*	POS (7)	3	4	6	0 *
	NEG (43)	0	43	0	38
*S. aureus*	POS (4)	3	1	1	0 **
	NEG (46)	0	46	0	40
*K. pneumoniae*	POS (2)	0	2	1	1
	NEG (48)	0	48	0	42
*A. baumannii*	POS (2)	0	2	0	1 *
	NEG (48)	0	48	0	42
*P. aeruginosa*	POS (2)	1	1	1	0 *
	NEG (48)	0	48	0	42
*E. faecium*	POS (0)	0	0	0	0
	NEG (50)	0	50	0	44
*Fusobacterium necrophorum*	POS (0)	0	0	0	0
	NEG (50)	1	49	0	44
*S. epidermidis*	POS (0)	0	0	0	0
	NEG (50)	1	49	0	44

* in one patient no culture was performed, ** in three patients no culture was performed; no multi-drug resistance bacteria were detected.

## Data Availability

All data generated or analysed during this study are included in this published article and its Supplementary Information Files.

## Supplementary Materials

The following supporting information can be downloaded at: https://www.mdpi.com/article/10.3390/microorganisms12050967/s1.

## References

[1] Buehler S.S., Madison B., Snyder S.R., Derzon J.H., Cornish N.E., Saubolle M.A., Weissfeld A.S., Weinstein M.P., Liebow E.B., Wolk D.M. (2016). Effectiveness of Practices To Increase Timeliness of Providing Targeted Therapy for Inpatients with Bloodstream Infections: A Laboratory Medicine Best Practices Systematic Review and Meta-analysis. Clin. Microbiol. Rev..

[2] Cohen J., Vincent J.L., Adhikari N.K.J., Machado F.R., Angus D.C., Calandra T., Jaton K., Giulieri S., Delaloye J., Opal S. (2015). Sepsis: A roadmap for future research. Lancet Infect. Dis..

[3] Kumar A., Ellis P., Arabi Y., Roberts D., Light B., Parrillo J.E., Dodek P., Wood G., Kumar A., Simon D. (2009). Initiation of inappropriate antimicrobial therapy results in a fivefold reduction of survival in human septic shock. Chest.

[4] Laupland K.B., Church D.L. (2014). Population-Based Epidemiology and Microbiology of Community-Onset Bloodstream Infections. Clin. Microbiol. Rev..

[5] Timsit J.F., Ruppé E., Barbier F., Tabah A., Bassetti M. (2020). Bloodstream infections in critically ill patients: An expert statement. Intensive Care Med..

[6] Faron M.L., Buchan B.W., Ledeboer N.A. (2017). Matrix-Assisted Laser Desorption Ionization-Time of Flight Mass Spectrometry for Use with Positive Blood Cultures: Methodology, Performance, and Optimization. J. Clin. Microbiol..

[7] Banerjee R., Teng C.B., Cunningham S.A., Ihde S.M., Steckelberg J.M., Moriarty J.P., Shah N.D., Mandrekar J.N., Patel R. (2015). Randomized Trial of Rapid Multiplex Polymerase Chain Reaction–Based Blood Culture Identification and Susceptibility Testing. Clin. Infect. Dis..

[8] Dunbar S.A., Gardner C., Das S. (2022). Diagnosis and Management of Bloodstream Infections With Rapid, Multiplexed Molecular Assays. Front. Cell. Infect. Microbiol..

[9] Bach K., Edel B., Höring S., Bartoničkova L., Glöckner S., Löffler B., Bahrs C., Rödel J. (2022). Performance of the eazyplex^®^ BloodScreen GN as a simple and rapid molecular test for identification of Gram-negative bacteria from positive blood cultures. Eur. J. Clin. Microbiol. Infect. Dis..

[10] Parize P., Muth E., Richaud C., Gratigny M., Pilmis B., Lamamy A., Mainardi J.-L., Cheval J., de Visser L., Jagorel F. (2017). Untargeted next-generation sequencing-based first-line diagnosis of infection in immunocompromised adults: A multicentre, blinded, prospective study. Clin. Microbiol. Infect..

[11] Blauwkamp T.A., Thair S., Rosen M.J., Blair L., Lindner M.S., Vilfan I.D., Kawli T., Christians F.C., Venkatasubrahmanyam S., Wall G.D. (2019). Analytical and clinical validation of a microbial cell-free DNA sequencing test for infectious disease. Nat. Microbiol..

[12] Clancy C.J., Pappas P.G., Vazquez J., Judson M.A., Kontoyiannis D.P., Thompson G.R., Garey K.W., Reboli A., Greenberg R.N., Apewokin S. (2018). Detecting Infections Rapidly and Easily for Candidemia Trial, Part 2 (DIRECT2): A Prospective, Multicenter Study of the T2Candida Panel. Clin. Infect. Dis..

[13] Mylonakis E., Clancy C.J., Ostrosky-Zeichner L., Garey K.W., Alangaden G.J., Vazquez J.A., Groeger J.S., Judson M.A., Vinagre Y.-M., Heard S.O. (2015). T2 magnetic resonance assay for the rapid diagnosis of candidemia in whole blood: A clinical trial. Clin. Infect. Dis..

[14] Seitz T., Holbik J., Hind J., Gibas G., Karolyi M., Pawelka E., Traugott M., Wenisch C., Zoufaly A. (2022). Rapid Detection of Bacterial and Fungal Pathogens Using the T2MR versus Blood Culture in Patients with Severe COVID-19. Microbiol. Spectr..

[15] Mylonakis E., Zacharioudakis I.M., Clancy C.J., Hong Nguyen M., Pappas P.G. (2018). Efficacy of T2 Magnetic Resonance Assay in Monitoring Candidemia after Initiation of Antifungal Therapy: The Serial Therapeutic and Antifungal Monitoring Protocol (STAMP) Trial. J. Clin. Microbiol..

[16] Muñoz P., Vena A., Machado M., Gioia F., Martínez-Jiménez M.C., Gómez E., Muñoz P., Martínez-Jiménez M.C., Gómez E., Origüen J. (2018). T2Candida MR as a predictor of outcome in patients with suspected invasive candidiasis starting empirical antifungal treatment: A prospective pilot study. J. Antimicrob. Chemother..

[17] Bilir S.P., Ferrufino C.P., Pfaller M.A., Munakata J. (2015). The economic impact of rapid Candida species identification by T2Candida among high-risk patients. Future Microbiol..

[18] Hong Nguyen M., Clancy C.J., William Pasculle A., Pappas P.G., Alangaden G., Pankey G.A., Schmitt B.H., Rasool A., Weinstein M.P., Widen R. (2019). Performance of the T2Bacteria Panel for Diagnosing Bloodstream Infections: A Diagnostic Accuracy Study. Ann. Intern. Med..

[19] AWMF Leitlinienregister Kalkulierte parenterale Initialtherapie Bakterieller Erkrankungen bei Erwachsenen—Update 2018. https://register.awmf.org/de/leitlinien/detail/082-006.

[20] Jordana-Lluch E., Rivaya B., Marcó C., Giménez M., Quesada M.D., Escobedo A., Batlle M., Martró E., Ausina V. (2017). Molecular diagnosis of bloodstream infections in onco-haematology patients with PCR/ESI-MS technology. J. Infect..

[21] De Angelis G., Posteraro B., De Carolis E., Menchinelli G., Franceschi F., Tumbarello M., De Pascale G., Spanu T., Sanguinetti M. (2018). T2Bacteria magnetic resonance assay for the rapid detection of ESKAPEc pathogens directly in whole blood. J. Antimicrob. Chemother..

[22] Caliendo A.M., Gilbert D.N., Ginocchio C.C., Hanson K.E., May L., Quinn T.C., Tenover F.C., Alland D., Blaschke A.J., Bonomo R.A. (2013). Better tests, better care: Improved diagnostics for infectious diseases. Clin. Infect. Dis..

[23] Tafelski S., Nachtigall I., Adam T., Bereswill S., Faust J., Tamarkin A., Trefzer T., Deja M., Idelevich E.A., Wernecke K.-D. (2015). Randomized controlled clinical trial evaluating multiplex polymerase chain reaction for pathogen identification and therapy adaptation in critical care patients with pulmonary or abdominal sepsis. J. Int. Med. Res..

[24] Gies F., Tschiedel E., Felderhoff-Müser U., Rath P.M., Steinmann J., Dohna-Schwake C. (2016). Prospective evaluation of SeptiFast Multiplex PCR in children with systemic inflammatory response syndrome under antibiotic treatment. BMC Infect. Dis..

[25] Box M.J., Sullivan E.L., Ortwine K.N., Parmenter M.A., Quigley M.M., Aguilar-Higgins L.M., MacIntosh C.L., Goerke K.F., Lim R.A. (2015). Outcomes of rapid identification for gram-positive bacteremia in combination with antibiotic stewardship at a community-based hospital system. Pharmacotherapy.

[26] Felsenstein S., Bender J.M., Sposto R., Gentry M., Takemoto C., Bard J.D. (2016). Impact of a Rapid Blood Culture Assay for Gram-Positive Identification and Detection of Resistance Markers in a Pediatric Hospital. Arch. Pathol. Lab. Med..

[27] Eby J.C., Richey M.M., Platts-Mills J.A., Mathers A.J., Novicoff W.M., Cox H.L. (2018). A Healthcare Improvement Intervention Combining Nucleic Acid Microarray Testing With Direct Physician Response for Management of *Staphylococcus aureus* Bacteremia. Clin. Infect. Dis..

[28] Weng T.P., Lo C.L., Lin W.L., Lee J.C., Li M.C., Ko W.C., Lee N.Y. (2023). Integration of antimicrobial stewardship intervention with rapid organism identification improve outcomes in adult patients with bloodstream infections. J. Microbiol. Immunol. Infect..

[29] Cavalieri S.J., Kwon S., Vivekanandan R., Ased S., Carroll C., Anthone J., Schmidt D., Baysden M., Destache C.J. (2019). Effect of antimicrobial stewardship with rapid MALDI-TOF identification and Vitek 2 antimicrobial susceptibility testing on hospitalization outcome. Diagn. Microbiol. Infect. Dis..

[30] Wang C., Huang Z., Li W., Fang X., Zhang W. (2020). Can metagenomic next-generation sequencing identify the pathogens responsible for culture-negative prosthetic joint infection?. BMC Infect. Dis..

[31] Voigt C., Silbert S., Widen R.H., Marturano J.E., Lowery T.J., Ashcraft D., Pankey G. (2020). The T2Bacteria Assay Is a Sensitive and Rapid Detector of Bacteremia That Can Be Initiated in the Emergency Department and Has Potential to Favorably Influence Subsequent Therapy. J. Emerg. Med..

[32] (2021). T2 Biosystems 101 Hartwell Avenue Lexington M 02421 P +1-781-457-1200 F +1-781-357-3080. T2Resistance Panel T2 Biosystems. https://www.t2biosystems.com/products-technology/pipeline/t2resistance-panel/.

[33] Nguyen M.H., Clancy C.C., Pasculle W., Pappas P., Alangaden G., Pankey G.A., Schmitt B.H., Rasool A., Weinstein M.B., Widen R. (2020). In-depth analysis of T2Bacteria positive results in patients with concurrent negative blood culture: A case series. BMC Infect. Dis..

