# Supplementary material for: Point-of-Care Method T2Bacteria®Panel Enables a More Sensitive and Rapid Diagnosis of Bacterial Blood Stream Infections and a Shorter Time until Targeted Therapy than Blood Culture
Source: Microorganisms. 2024 May 11;12(5):967. doi: 10.3390/microorganisms12050967 (PMC11124434; doi:10.3390/microorganisms12050967)
Supplement: Supplementary file 1 [file microorganisms-12-00967-s001.zip › microorganisms-2987415-supplementary.pdf]

SUPPLEMENT S1

INCLUSION CRITERIA

1. Patient showed signs of systemic infection (meeting at least two criteria; one in each section):
   - Temperature ≥ 38°C

   and

   - Heart Rate > 90/min
   - Systolic Blood Pressure < 100mmHg
   - Respiratory Rate ≥ 22/min
   - GCS<15

AND

2. Patients showed bacteria specific clinical signs/symptoms of infection with an ESKAPE pathogen (at least one criterion):
   - positive urine test strip (Leukocyturia)
   - acute bacterial skin and skin structure infections (ABSSSI)
   - signs of abscesses in imaging procedure
   - acute backpain without trauma and clinical suspected spondylodiscitis
   - recent intravenous drug abuse
   - endocarditis
   - presence of intravascular devices
   - presence of peritoneal dialysis
   - previous evidence of colonization with ESKAPE organisms
   - intraabdominal infection

EXCLUSION CRITERIA

- younger than 18 years
- presence of pneumonia (including clinical suspicion of pneumonia)
- presence of meningitis (including clinical suspicion of meningitis)
- presence ears, nose and throat (ENT) infection (including clinical suspicion of ENT infection)
- presence of or suspicion of colitis
- presence of Influenza or other viral disease explaining the symptoms
- persons who are incapable of giving consent

SUPPLEMENT S2

Defined targeted therapy depending on the found pathogen.

**Pathogen**	**Targeted therapy**	**Targeted therapy if penicillin allergy exists**
Enterococcus faecium	Linezolid, Vancomycin, Daptomycin	
Staphylococcus aureus	Isoxazolylpenicillin, Cephalosporine 1st Generation	
Klebsiella pneumoniae	Cephalosporine 3rd Generation,	Ciprofloxacin/Levofloxacin
Acinetobacter baumannii	Meropenem	-
Pseudomonas aeruginosa	Piperacillin/Tazobactam, Ceftazidim, Cefepime	Aztreonam
Escherichia coli	Cephalosporine 2nd or 3rd Generation	-
